# *ALKBH5* inhibitors as a potential treatment strategy in heart failure—inferences from gene expression profiling

**DOI:** 10.3389/fcvm.2023.1194311

**Published:** 2023-07-31

**Authors:** Sumra Komal, Atia Gohar, Saad Althobaiti, Ishtiaq Ahmad Khan, Liu-Gen Cui, Li-Rong Zhang, Sheng-Na Han, Muhammad Shakeel

**Affiliations:** ^1^ Department of Pharmacology, School of Basic Medical Sciences, Zhengzhou University, Zhengzhou, China; ^2^ Dow Institute for Advanced Biological and Animal Research, Dow University of Health Sciences, Karachi, Pakistan; ^3^ King Faisal Medical Complex (KFMC), Taif, Saudi Arabia; ^4^ Jamil-ur-Rahman Center for Genome Research, Dr. Panjwani Center for Molecular Medicine and Drug Research, International Center for Chemical and Biological Sciences, University of Karachi, Karachi, Pakistan

**Keywords:** AlkB homolog 5, epitranscriptomics, N6-methyladenosine, transcriptome, heart failure

## Abstract

Heart Failure (HF) is a complex clinical syndrome in which the heart is unable to provide enough blood flow to meet metabolic needs and lacks efficient venous return. HF is a major risk factor for morbidity and mortality with cardiovascular diseases globally. Despite enormous research, the molecular markers relevant to disease prognosis and management remain not well understood. Here, we analyzed the whole transcriptomes of 18 failing hearts and 15 non-failing hearts (predominantly of Caucasian origin), by applying the standard *in silico* tools. The analyses revealed novel gene-markers including *ALKBH5* of mRNA demethylation and *KMT2E* of histone modification processes, significantly over-expressed in the HF compared with the non-failing hearts (FDR < 0.05). To validate the over-expression of *ALKBH5*, we determined the global m^6^A level in hypoxic H9c2 cells using a dot blot assay. The global m^6^A level was found markedly lower in the hypoxic H9c2 cells than in the control cells. Additionally, the expression of *ALKBH5* in the H9c2 cells was quantified by the qPCR and found to be 1.18 times higher at 12 h (*p* < 0.05), and 1.67 times higher at 24 h of hypoxia (*p* < 0.01) compared with the control cells, indicating a likely role of *ALKBH5* in the failing cardiac cells. Furthermore, we identified several compounds through the virtual screening of 11,272 drug-like molecules of the ZINC15 database to inhibit the *ALKBH5* in a molecular docking process. Collectively, the study revealed novel markers potentially involved in the pathophysiology of HF and suggested plausible therapeutic molecules for the management of the disease.

## 1. Introduction

Heart failure (HF) is leading in terms of prevalence and hospitalization worldwide. Cardiovascular diseases (CVDs) are associated with significantly higher morbidity, mortality, and healthcare expenditures, particularly among older adults (1). Despite improvements in the healthcare system and advancements in modern medicine, the prognosis of HF has not improved over the last decades, suggesting that several aspects of cardiac pathophysiology remain unresolved (2). Therefore, comprehension of the underlying mechanisms in HF can play a major role in disease prevention and management.

Epidemiological findings suggest that alcohol, obesity, smoking, hypertension, and coronary artery disease (CAD) are the risk factors for HF (3, 4). At molecular level, the recent research suggests that ventricular remodeling, compensatory mechanisms, inflammatory signaling, fibrosis, oxidative stress, mitochondrial dysfunction, and epigenetic modifications, including RNA transcript modifications, are significantly associated with HF progression and predict HF outcomes (5). Ventricular remodeling, which is a modification of the structure and function of the heart after injury to the myocardium, involves excitation-contraction coupling, Ca^2+^ handling, changes in energetic metabolism, contractile proteins or their regulatory elements, and cytoskeleton components (6). Compensatory mechanisms, such as activation of the adrenergic nervous system (Beta-Blocker Evaluation of Survival Trial Investigators, 2001) and renin-angiotensin-aldosterone system (RAAS) (7), also contribute to the onset of HF. Cardiac fibrosis is a determinant of HF outcome and is promoted by irregular deposition of extracellular matrix (ECM) proteins in the cardiac interstitium (8). Cardiac fibrosis leads to diastolic and systolic dysfunction of the left ventricle (9). Cardiac inflammation occurs primarily in response to cardiac injury such as ischemia. In short-term cardiac injury, activation of nuclear factor kappa B (NF-κB) induces a cardio-protective role (10). However, long-term heart damage activates the release of several proinflammatory cytokines and chemokines leading to HF (11). The activation of cell surface death receptors, such as Fas/FasL, and consequent programmed cell death are also found in failing hearts (12). Oxidative stress is also associated with ventricular dysfunction, with increased production of reactive oxygen species (ROS) stimulated by aldosterone, which is produced by the RAAS, observed in HF (13).

In addition to the afore-mentioned etiologies of HF, several studies have shown in the recent past that dysregulation of the epitranscriptomic modifiers such as mRNA N6-methyladenosine (m^6^A) writers (*METTL3*, and *METTL14*), readers (*YTHDF1*, *YTHDF2*, and *YTHDF3*), and erasers (*FTO, ALKBH5*), which affect the stability, splicing, translation efficiency, and degradation of the mRNA (14–18), have plausible roles in the pathophysiology of HF (19). These findings also suggest the potential use of these genes as new biomarkers in the HF. In this context, two m^6^A demethylases, fat mass and obesity–associated protein (*FTO*) and AlkB homolog 5 (*ALKBH5*) have been given particular emphasis in the translational cardiovascular research. The expression of *FTO* regulates the cardiac function and has been implicated in HF (20). However, the role of *ALKBH5* demethylase has been less studied in HF. Previously, *ALKBH5* inhibited apoptosis and promoted autophagy in nucleus pulposus cells by demethylating the FIP200 mRNA (21). So, there is a need to explore the involvement of *ALKBH5* in HF patients.

The advent of robust approaches, such as whole transcriptome analysis, has enabled us to explore deeply and discover new therapeutic targets involved in the pathophysiology of HF. In this study, we aimed to analyze the whole transcriptomes of failing hearts of different age groups, in comparison with non-failing hearts to identify distinct gene expression profiles and their roles in the disease. Our findings revealed some novel gene markers of epitranscriptomic modification, and thus, suggested potential pharmacological targets in HF. Additionally, we performed molecular docking of over 11,000 compounds from the ZINC15 database to explore potential molecules for the development of new drugs.

## 2. Methodology

### 2.1. Data collection

The RNA-seq data of human left ventricles and related metadata of 18 HF patients and 15 healthy individuals were retrieved from the Gene Expression Omnibus (GEO, https://www.ncbi.nlm.nih.gov/geo). The study cohort related to the gene expression omnibus studies GSE57344, GSE147236, and GSE161472. The metadata was normalized to get uniform information about the samples. The RNA-seq data was fetched with the SRA Toolkit using the “SRR” IDs of the sample and then converted into the fastq format using the fastq-dump tool of the SRA Toolkit (22).

### 2.2. Bioinformatics analysis

The fastq files were assessed for the Phred quality scores using the FastQC tool v0.11.9 (23). The low-quality reads were filtered out using the Trimmomatic tool v0.40 (24). For the alignment of short sequence reads with reference, the STAR 2.7.10a pipeline (25) of The Cancer Genome Atlas (TCGA) was employed. The sequencing reads were aligned to the hg38 assembly of the human reference genome (ftp://hgdownload.soe.ucsc.edu/goldenPath). In the STAR analysis, first, the reference genome was indexed using the “genomeGenerate” module, followed by Alignment First Pass, Intermediate Index Generation, and Alignment Second Pass. The number of aligned reads in each gene was counted in the resultant BAM file using the featureCounts tool. For this, the reference genome-compatible genes’ coordinates (gtf) were obtained from the GENCODE database (https://www.gencodegenes.org/).

### 2.3. Comparison of genes expression changes

For determining the global gene expression profiling and differential gene expression, the raw read count data were transformed into fragments per kilo per million (FPKM) values using the R package (v.4.1.2) by applying the formula:

$$\mathrm{FPK}M_{i}=\frac{q_{i}}{(l_{i}/10^{3})*\left(\sum {}_{j}q_{j}/10^{6}\right)}$$

where, for the FPKM of the *i*th feature (gene), *q_i_* is the raw read count of the gene, *l_i_* is the length of the gene, and ∑*_j_q_j_* corresponds to the total number of mapped fragments. The BioConductor package EdgeR was employed to perform the downstream analysis for determining the differential genes expression. First, the data was normalized by applying a weighted trimmed mean of *M* values (TMM) method. Then Fisher's exact test was applied to conduct a pairwise comparison of genes expression between the normal and HF samples for each gene. The edgeR's function “topTags” which employs Benjamini-Hochberg method was used to apply multiple corrections and to minimize the false discovery rate (FDR), also termed as the adjusted *p*-values. The FDR < 0.05 was considered significant for designating a gene as differentially expressed. Further, the unsupervised hierarchal clustering was performed to find out the distinct groups based on the expression profiles. To further extrapolate and to find the statistical significance among the groups identified in the hierarchal clustering, the Kruskal-Wallis test was performed. The expression profiles were further evaluated by performing principal components analysis (PCA). The unsupervised hierarchal clustering, Kruska-Wallis test, PCA, and the data visualizations were carried out by using the R-4.0.3 statistical package.

### 2.4. Gene annotation and gene/protein regulatory network

To determine the biological processes, gene set enrichment analysis and functional protein association networks related to the prioritized genes, the online STRING database (v.11.5) was used (https://string-db.org/). The protein-protein interactions (PPIs) were determined within the over-expressed and under-expressed genes. The protein-protein interactions with PPI score ≥0.4 were considered. The whole bioinformatics pipeline has been submitted to the GitHub repository https://github.com/Shakeel211/RNA-seq.

### 2.5. H9c2 cell culture and hypoxia model

Given the recent focus of biomedical research on epitranscriptomics in HF (17, 26), we selected one gene *ALKBH5* (an RNA demethylase and epitranscriptomic marker) for validation in the cardiac cell line. We constructed the hypoxia model using the H9c2 (rat embryonic ventricle) cell line, as described in our previous study (27). The H9c2 cell line was purchased from the Type Culture Collection of the Chinese Academy of Sciences (Shanghai, China). An anaerobic workstation (AW400SG/TG; Electrotek) was used to prepare the cell hypoxia model. H9c2 cells were placed in the transfer chamber, exposed to high-purity N_2_, and then transferred into the anaerobic working chamber where mixed gas consisting of 10% H_2_, 10% CO_2_, and 80% N_2_ was introduced. Dulbecco's modified Eagle medium (DMEM, Corning Inc., Corning, NY, USA) supplemented with 10% fetal bovine serum (FBS) and 1% penicillin/streptomycin) was used for cell culture. When the cells reached 80%–90% confluency, the medium was replaced with serum-free DMEM medium, and cells were cultured for 12, and 24 h under hypoxia conditions, respectively.

### 2.6. m^6^A dot blot assay

To quantify m^6^A level in the hypoxia-induced and normal H9c2 cells, 500 ng RNAs were denatured by heating at 65°C for 5 min and spotted onto a nylon membrane (Sigma, USA) with a Bio-Dot apparatus (Bio-Rad, USA). The membrane was ultraviolet (UV) crosslinked, blocked, incubated with m^6^A antibody overnight at 4°C, and then incubated with HRP-conjugated anti-mouse IgG. After extensive washing, the membrane was visualized by the chemiluminescence system (Bio-Rad, USA). After visualization, the membrane was stained with 0.02% methylene blue (MB) in 0.3 M sodium acetate buffer (pH 5.2) to ensure consistency in the RNA amount of the samples on the membrane.

### 2.7. RNA extraction and quantitative PCR (qPCR) analysis, and western blotting

For performing the quantitative real-time PCR (qPCR) of *ALKBH5*, total RNA was isolated by using the TRIzol reagent (Roche, Germany), and reverse transcribed by using the PrimeScript RT Reagent Kit (Cat#RR047A, Takara Bio, China) following the recommended protocol of the manufacturer. The resultant cDNA was subject to qPCR by using the SYBR Green Master mix (Thermo Fisher Scientific, United States) on the 7,500 Fast Real-Time PCR system (Applied Biosystems; Thermo Fisher Scientific, United States). In the qPCR, the following thermocycling conditions were used: holding at 50°C for 2 min; pre-denaturation at 95°C for 2 min, followed by 40 cycles of 15 s at 95°C and 1 min at 60°C. For the melt curve, 95°C for 15 s, 60°C for 1 min, 95°C for 15 s, and 60°C for 15 s. were applied. The *ALKBH5* mRNA levels were normalized to *GAPDH* levels. Following primers were used in the qPCR reaction: *ALKBH5* Forward: 5′- CCCGAGGGCTTCGTCAACA-3′; Reverse: 5′-CGACACCCGAATAGGCTTGA-3′; *GAPDH* Forward: 5′-TCCCTCAAGATTGTCAGCAA-3′; Reverse: 5′-AGATCCACAACGGATACATT-3′. The relative expression was calculated using the 2−ΔΔCT method. For determining the statistical significance, one-way ANOVA was used. For the western blot analysis, total protein from H9c2 cells was collected using RIPA lysis buffer (#R0030, Solarbio,Beijing, China) plus protease inhibitor cocktail (#HY-K0010, MedChemExpress, New Jersey, USA). The membranes were incubated with primary antibodies against ALKBH5 (1:2000; #16837-1-AP, Proteintech, Wuhan, China), and GAPDH (1:10000; #60004-1-Ig, Proteintech) at 4°C overnight, followed by alkaline phosphatase-conjugated affinipure goat anti-rabbit IgG (H+L) (1:10000; Proteintech) for 2 h at 37°C. The blots were analyzed and quantified using ImageJ analysis software.

### 2.8. Virtual screening of drug-like compounds through the molecular docking

A virtual screening of 11,272 drug-like compounds from the ZINC15 database (www.zinc15.docking.org) was performed against the druggable active sites (putative targets) of the *ALKBH5* protein (PDB ID 4NJ4) using the Molecular Operating Environment (MOE v2016.11) software (28). Prior to the docking, the druggable target sites (pockets) in the *ALKBH5* were identified through the DoGSiteScorer server (https://proteins.plus). The MOE predicts accurately the binding affinities of the ligands with the protein through a predefined algorithm by scoring different docking orientations between the receptor and ligand in a ligand receptor interaction and records affinities of the best fit poses of the ligands. The docking was performed according to the protocol as described by the authors (28). The parameters used were as follows: Placement = Triangle matcher, Refinement = Induced fit, Scoring 1 = London dG kcal/mol, Scoring 2 = GBVI/WSA dG kcal/mol, Poses = 30, and Retain Poses = 5.

### 2.9. ADMET profiling of the lead compounds

The top 10 hits which passed the Lipinski's drug-like test and had minimum values of binding energies were selected as suitable inhibitors for performing the pharmacokinetics analysis including the absorption, distribution, metabolism, excretion and toxicity (ADMET) analysis. The ADMET analysis and other physicochemical parameters such as skin permeation were determined using the online ADMET prediction server (http://lmmd.ecust.edu.cn/admetsar2) and Swiss ADME (http://www.swissadme.ch/), respectively to validate the parameters for the suitable drug/binding candidates. Before performing the docking analysis, the structure of each ligand was optimized by calculating charges, applying force field (MMFF94x) and energy minimization.

### 2.10. Statistical analysis

The data are presented as means ± standard deviation (SD). Statistical significance was assessed using the unpaired student's *t*-test for experiments. *p*-value < 0.05 were considered statistically significant (*p < 0.05, **p < 0.01).

## 3. Results

### 3.1. Cohort characteristics

Characteristics of the study participants including the non-heart failing (NF) controls and heart failure (HF) patients are listed in Table 1. The study cohort predominantly comprised Caucasians (73%), whereas 15% of the participants were African-American, and 12% were Hispanic. The male:female ratio in the non-heart failing samples was 1.5, and in the heart failing sample was 1.57. The age of the non-heart failing individuals was 1–77 years (average 46.6 years, median 50 years), and of the HF patients was 16–68 years (average 39.38 years, median 38.5 years). The patients presented with HF due to cardiomyopathies.

**Table 1:** Characteristics of the study cohort. The non-failing healthy hearts were included as controls, whereas, the failing hearts were included as diseases samples.

SRA ID	Bio-sample	Disease	Sex	Age (Y)	Ethnicity	Sample code
Healthy controls						
SRR11351704	SAMN14405137	Non-failing heart	Male	62	Caucasian	norm1
SRR11351705	SAMN14405136	Non-failing heart	Male	47	Caucasian	norm2
SRR11351706	SAMN14405135	Non-failing heart	Female	76	Caucasian	norm3
SRR1272186	SAMN02746604	Non-failing heart	Female	57	White/Caucasian	norm4
SRR1272187	SAMN02746607	Non-failing heart	Male	1	White/Caucasian	norm5
SRR1272188	SAMN02746605	Non-failing heart	Male	52	White/Caucasian	norm6
SRR13057971	SAMN16793894	Non-failing heart	Male	53	Caucasian	norm7
SRR13057972	SAMN16793893	Non-failing heart	Female	27	Hispanic	norm8
SRR13057973	SAMN16793892	Non-failing heart	Male	47	African-American	norm9
SRR13057974	SAMN16793891	Non-failing heart	Male	54	Caucasian	norm10
SRR13057975	SAMN16793890	Non-failing heart	Female	44	Caucasian	norm11
SRR13057976	SAMN16793889	Non-failing heart	Female	28	African-American	norm12
SRR13057977	SAMN16793888	Non-failing heart	Male	50	Hispanic	norm13
SRR13057978	SAMN16793835	Non-failing heart	Male	48	Caucasian	norm14
SRR13057979	SAMN16793826	Non-failing heart	Female	53	African-American	norm15
Heart failures						
SRR11351707	SAMN14405134	Heart failure	Male	20	Caucasian	card1
SRR11351708	SAMN14405133	Heart failure	Male	16	Hispanic	card2
SRR11351710	SAMN14405131	Heart failure	Female	26	Caucasian	card4
SRR13057981	SAMN16793833	HFrEF	Male	26	Caucasian	card8
SRR13057982	SAMN16793832	HFrEF	Male	22	African-American	card9
SRR13057983	SAMN16793831	HFrEF	Male	30	Caucasian	card10
SRR13057984	SAMN16793830	HFrEF	Male	28	Caucasian	card11
SRR13057989	SAMN16793824	HFrEF	Male	23	Caucasian	card16
SRR13057980	SAMN16793834	HFrEF	Male	37	African-American	card7
SRR13057985	SAMN16793829	HFrEF	Female	40	Caucasian	card12
SRR13057987	SAMN16793827	HFrEF	Female	50	Hispanic	card14
SRR13057990	SAMN16793823	HFrEF	Male	45	Caucasian	card17
SRR11351709	SAMN14405132	Heart failure	Male	54	Caucasian	card3
SRR1272190	SAMN02746608	Heart failure	Male	68	White/Caucasian	card5
SRR1272191	SAMN02746606	Heart failure	Female	59	White/Caucasian	card6
SRR13057986	SAMN16793828	HFrEF	Female	53	Caucasian	card13
SRR13057988	SAMN16793825	HFrEF	Female	55	Caucasian	card15
SRR13057991	SAMN16793822	HFrEF	Female	57	Caucasian	card18

Y, Years; HFrEF, Heart failure with reduced ejection fraction.

### 3.2. Transcriptome profiles of heart failure indicate the involvement of different signaling pathways

The transcriptomic profiling and differential gene expression was carried out schematically using the standard RNA-seq data analysis pipeline. The average FPKM was 9.5, whereas the median FPKM was found to be 4.1 (Supplementary Figure S1). The distribution of the FPKM values indicated a uniform trend, however, there were observable differences in the distribution in some bins (Supplementary Figure S2), indicating the likely differential expression of genes in these bins. For subsequent analysis, we selected the genes for which at least 50% of the samples had FPKM values of ≥0.5. This filtration resulted in 7,407 genes. Prior to the differential gene expression, a holistic view of the FPKM values was assessed, which indicated a mixed pattern of normal and HF samples (Supplementary Figure S3).

To determine the over-expressed and under-expressed genes in each sample, genes expression profiles were constructed. Taking FDR < 0.05 into account, there were 1,068 over-expressed genes (FDR < 0.05), and 924 under-expressed genes in the HF samples compared with the non-failing hearts (Supplementary Table S1). These included 498 over-expressed genes with a fold change (FC) ≥1.3 times higher in failing heart, and 413 under-expressed genes with FC ≤ 1.3 times less in failing heart.

**Figure 1:**
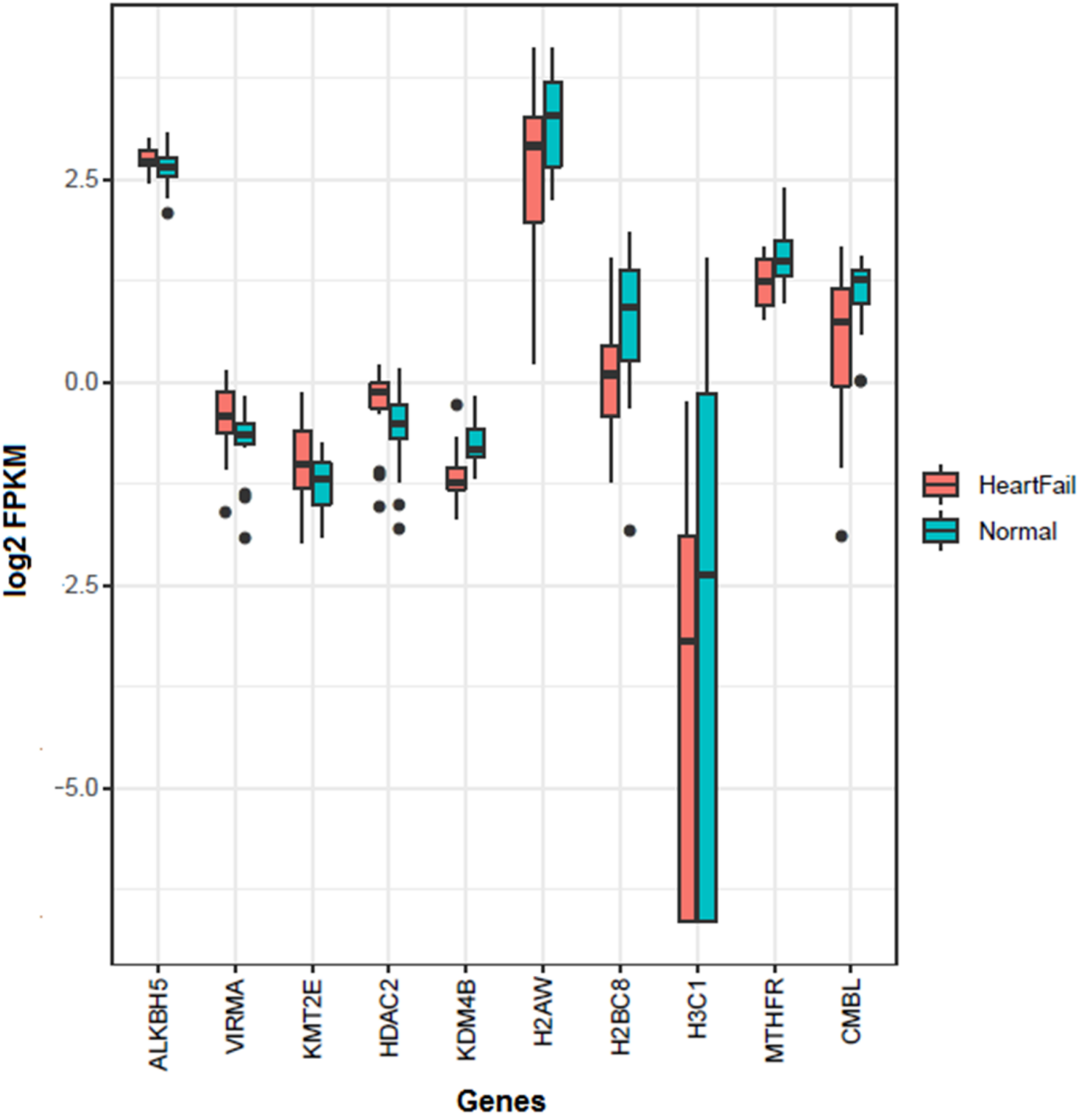
Significantly differentially expressed genes of m^6^A and histones methylation and demethylation in the heart failure compared with the normal hearts. Four genes (*ALKBH5*, *VIRMA*, *KMT2E*, and *HDAC2*) were over-expressed, whereas six genes (*KDM4B*, *H2AW*, *H2BC8*, *H3C1*, *MTHFR*, and *CMBL*) were down-expressed in the heart failures.

The gene set enrichment analysis of the over-expressed genes using the online STRING database (29) indicated multiple processes involved in the pathophysiology of HF (FDR < 0.05) (Supplementary Table S2A). These included 18 GO terms related to chemotaxis and immune cell migration, 14 terms related to regulation of cytoskeleton, 14 Gene Ontology (GO) terms related to protein folding, 5 terms related to various blood cell degranulation, and 3 terms related to platelet activation and migration. Among the Reactome pathways, the majority of the genes including *ANXA1, ANXA2, C3AR1, CCL2, CCL11, CD63, CXCL1, FSCN1, ITGB1, ITGB2, MAP2K3, PPIA, PPP2CB, RAP1B, TMEM30A*, and *TNFAIP3* were related with signaling by the interleukins and immune cells activation (Supplementary Table S3). Few genes including *ACTG1*, *ARRB2*, *FN1*, *NRAS*, and *RAP1B* were found related to JAK-STAT, MAPK, RAF1, BRAF, EPHB, and RAS signaling pathways. Notably, several genes were found associated with the platelets activation, signaling, degranulation, and aggregation, and included *ACTN1*, *ANXA5, AVPR1A, CALU, CAP1, CD63, CDC37L1, DGKD, F2R, FAM3C, FCER1G, FN1, GNG12, HSPA5, MANF, PDPN, PFN1, PPIA, PTPN1, RAP1B, SERPINE1, THBS1, TUBA4A,* and *YWHAZ*. Among these, five genes *CAP1, DGKD, F2R, FAM3C, and GNG12* have been prioritized for the first time in HF (Supplementary Figure S4). We further investigated the expression level of the epigenetic gene involved in m^6^A and histone methylation and demethylation. A significantly (FDR < 0.05) increased expression of *ALKBH5*, *VIRMA*, *KMT2E*, and *HDAC2*, whereas, reduced expression and *KDM4B*, *H2AW*, *H2BC8*, *H3C1*, *MTHFR*, and *CMBL* were found in the HF patients compared to the control group (Figure 1). Several protein-protein interactions (PPIs) among the over-expressed genes were also observed. These included 10 PPIs with very high confidence (PPI score ≥0.9), 11 PPIs with high confidence (PPI score 0.7–0.9), and 35 PPIs with moderate confidence (PPI score 0.4–0.7) (Supplementary Table S2B).

In addition, we compared the genes expression between the females and males heart failures to observe sex-wise distinct genes expression. This analysis highlighted 8 genes having lower expression in males compared with that in females. These genes included *FN1*, *PTPN1*, *ITGA3*, *F2R*, *MYH9*, *ITGB1*, *ACTG1*, and *ACTN1* (Supplementary Figure S5). The annotation and protein-protein interaction using the online STRING database showed that these genes were related with KEGG pathways Regulation of actin cytoskeleton (hsa04810), Focal adhesion (hsa04510), and PI3K-Akt signaling pathway (hsa04151).

### 3.3. *ALKBH5* expression, and global m^6^A methylation levels in the H9c2 cells

**Figure 2:**
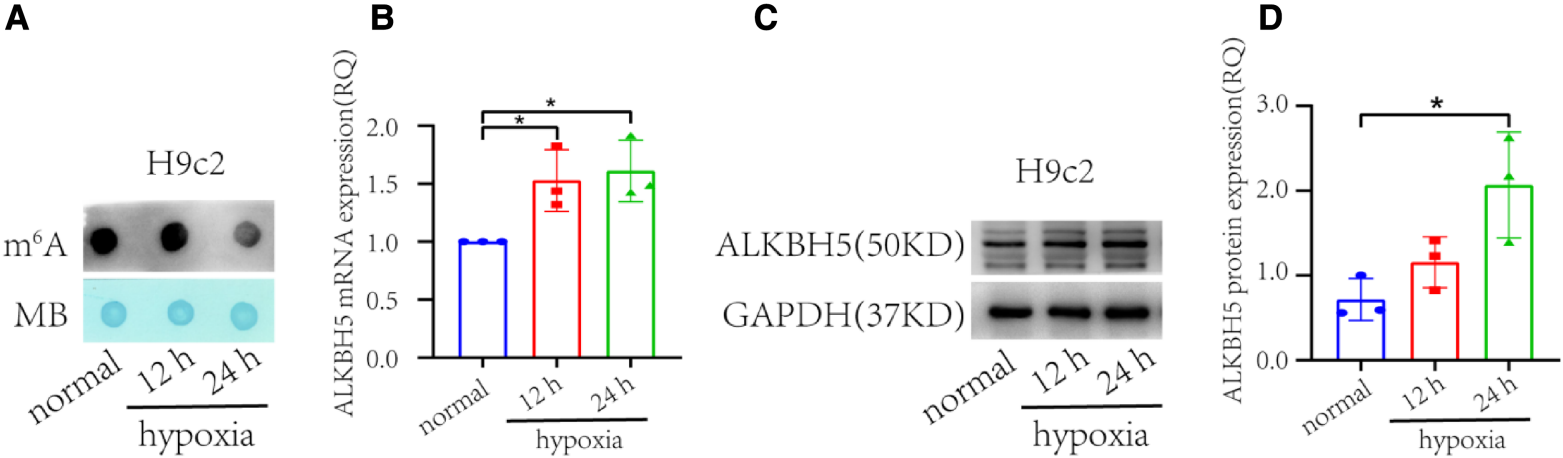
m^6^A abundance and *ALKBH5* expression in H9c2 cells under hypoxia and normal (non-hypoxic) conditions (*n* = 3 each). (**A**) Dot blot assay representing time dependent markedly lower global m^6^A in the H9c2 cells under hypoxia than in normal (non-hypoxic) condition. (**B**) *ALKBH5* gene expression in the H9c2 cell line under hypoxia, and normal conditions. (**C**) Western blot of ALKBH5 and GAPDH proteins in H9c2 cells. (**D**) The ALKBH5 protein expression in H9c2 cells inferred from the western blot using ImageJ. normal = H9c2 cells kept in non-hypoxic conditions; 12 h = H9c2 cells kept in hypoxic conditions for 12 h, 24 h = H9c2 cells kept under hypoxic conditions for 24 h. *p < 0.05, v.s. normal.

Out of the novel genes differentially expressed in the failing hearts, we selected *ALKBH5* to validate its expression in the H9c2 cells under hypoxia. The *ALKBH5* encodes an mRNA N6-methyladenosine dioxygenase which demethylates N(6)-methyladenosine (m^6^A) on RNAs. Given the RNA m^6^A demethylation role of *ALKBH5*, we determined the global m^6^A RNA methylation level in the H9c2 hypoxic and control cells using a dot blot assay (Figure 2A). In this analysis, time-dependent significant increase in *ALKBH5* expression was observed in the H9c2 cells under hypoxia compared with normal H9c2 cells (*p* < 0.05), where prolonged hypoxia caused higher expression (Figure 2B). To further validate, we evaluated the ALKBH5 protein level in H9c2 cells using the western blotting method. The expression of ALKBH5 protein was significantly higher in the H9c2 cells under 24 h of hypoxia (Figures 2C,D). These results showed that the decrease of m^6^A abundance may be caused by the increase of *ALKBH5* expression in hypoxic H9c2 cells.

### 3.4. Distinct expression profiles in age-dependent heart failure

**Figure 3:**
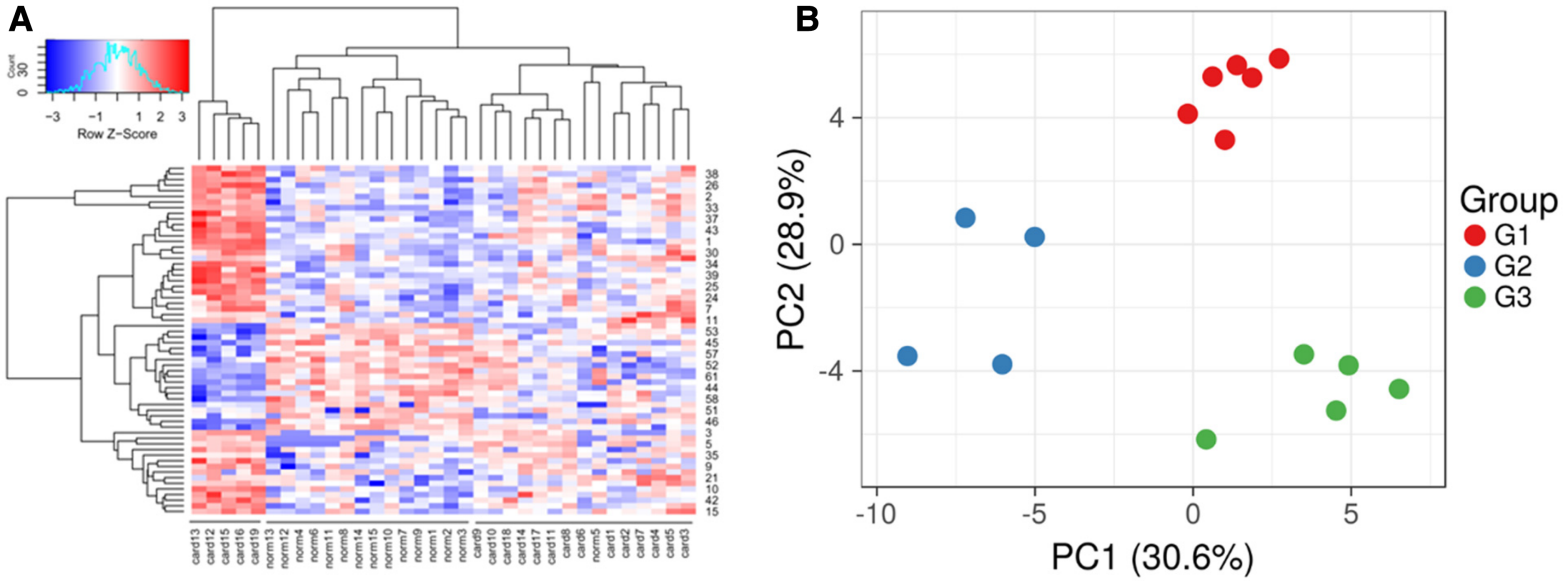
Expression profiles using unsupervised clustering. (**A**) The dendrogram shows three groups, including two groups of heart failure samples and one group of normal samples. (**B**) Divergence of the HF samples using principal components analysis. Three groups were observed (G1, G2, and G3), which were found to be age-related heart failure.

An unsupervised hierarchal clustering using the R package, revealed three distinct groups, one non-failing heart, and two HFs (Figure 3A). These indicated distinct expression profiles within the HF patients, indicating likely diverse mechanisms being involved in the HF. To further extrapolate this, a Kruskal-Wallis test was performed, which indicated diversity in the expression landscape among all HF samples (*p* < 0.001, df = 17). Furthermore, by performing principal components analysis (PCA) of the HF of different ages, three groups were identified (Figure 3B). The three HF groups observed in the PCA also exhibited significantly different expression profiles in the Kruskal-Wallis test (*p* < 0.01, df = 2). To further extrapolate, we identified 62 genes with ≥1.3 times higher expression and 191 genes having ≤1.3 times lower expression in group 1 patients with respect to group 2 and 3 patients (Supplementary Table S4). Group 1 comprised 6 patients (4 Caucasians, 1 African-American, and 1 Hispanic; 5 males and 1 female; an average age of 23.33 years), and we designated this group as the young-aged group. Likewise, in group 2, there were 4 patients (2 Caucasians, 1 African-American, and 1 Hispanic; 2 males and 2 females; with an average age of 43 years). The group was designated as the late-aged adult group. There were 57 differentially over-expressed genes and 9 genes that were less expressed in the Group 2 patients compared with the Group1 and Group3 patients (see Supplementary Table S5). The third group in the hierarchical clustering (Group 3) comprised 6 patients, all Caucasians (2 males, and 4 females), with an average age of 57.66 years, and was designated as the early-aged elder group. There were 215 differentially over-expressed genes and 193 under-expressed genes (see Supplementary Table S6).

### 3.5. Molecular docking and ADMET profiling

**Figure 4:**
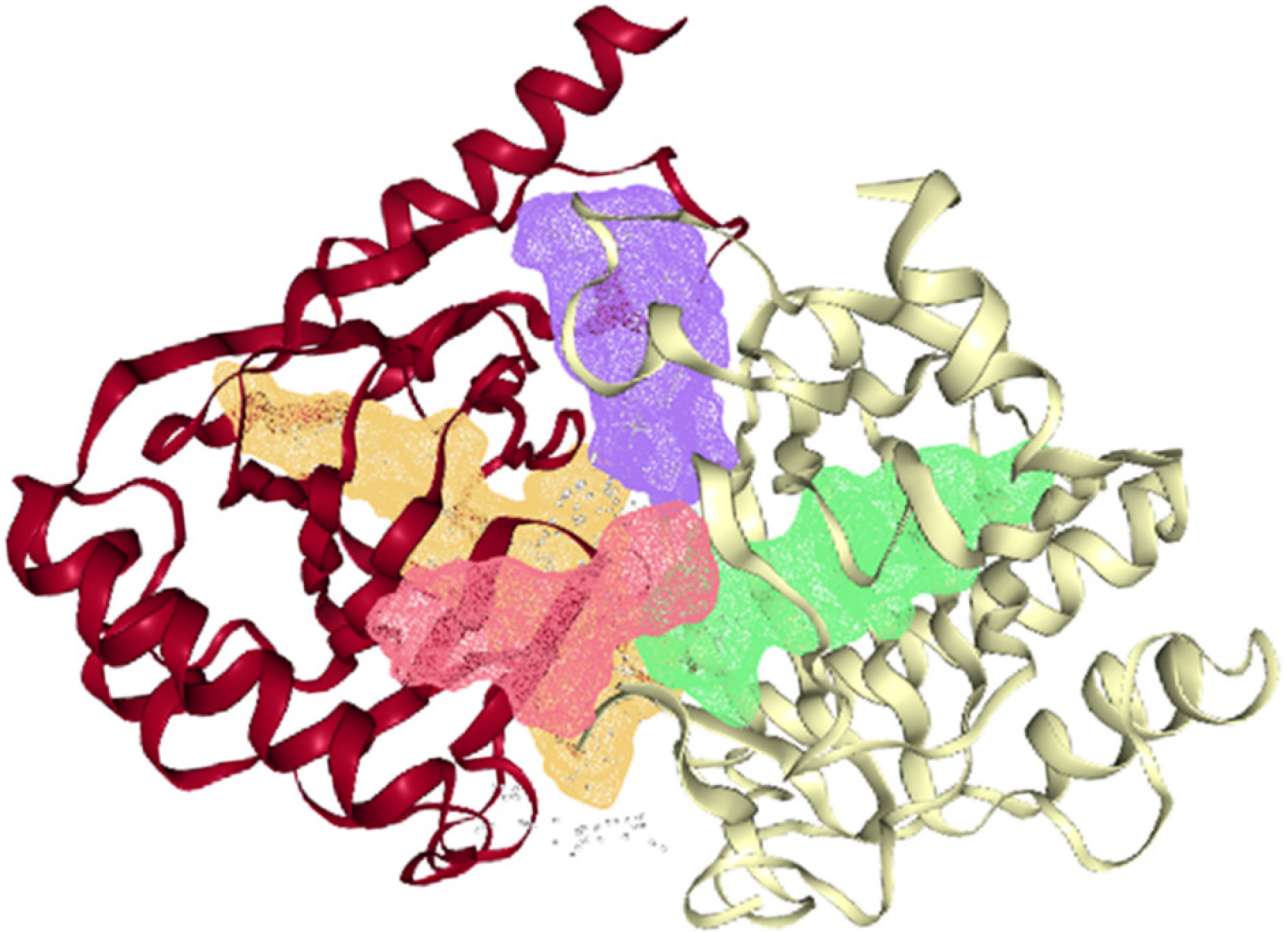
Four putative druggable pockets identified in the pdb structure of *ALKBH5* (4nj4) through the online DoGSiteScorer tool. The four pockets are represented by green (druggability score 0.85), violet (score 0.83), yellow (score 0.81), and red (score 0.7) colors inside the ribbons of the protein.

The *ALKBH5* protein structure (4NJ4) was downloaded from the RCSB PDB databank (https://www.rcsb.org/). Structurally, similar to the 2-oxoglutarate and iron-dependent oxygenase family members, *ALKBH5*_66–292_ has a double-stranded-helix core fold. An HXD…H motif (comprising residues His204, Asp206, and His266) and three water molecules octahedrally coordinate the active site metal. The *ALKBH5* has conserved active site residues and a nucleotide recognition lid as observed in other nucleic acid oxygenases (30). The energy minimized pdb structure of the protein was subjected to DoGSiteScorer server for identification of the putative active sites. The DoGSiteScorer identified 4 putative druggable pockets in the *ALKBH5* (Figure 4). The DoGSiteScorer is a tool to examine a protein's structure and to recognize pockets to compute the druggability of protein cavities. The druggability score predicted by the DoGSiteScorer tool ranges from 0 to 1. A value closer to 1 indicates a highly druggable protein cavity where the ligand will bind with high affinity i.e., minimum binding energy.

**Figure 5:**
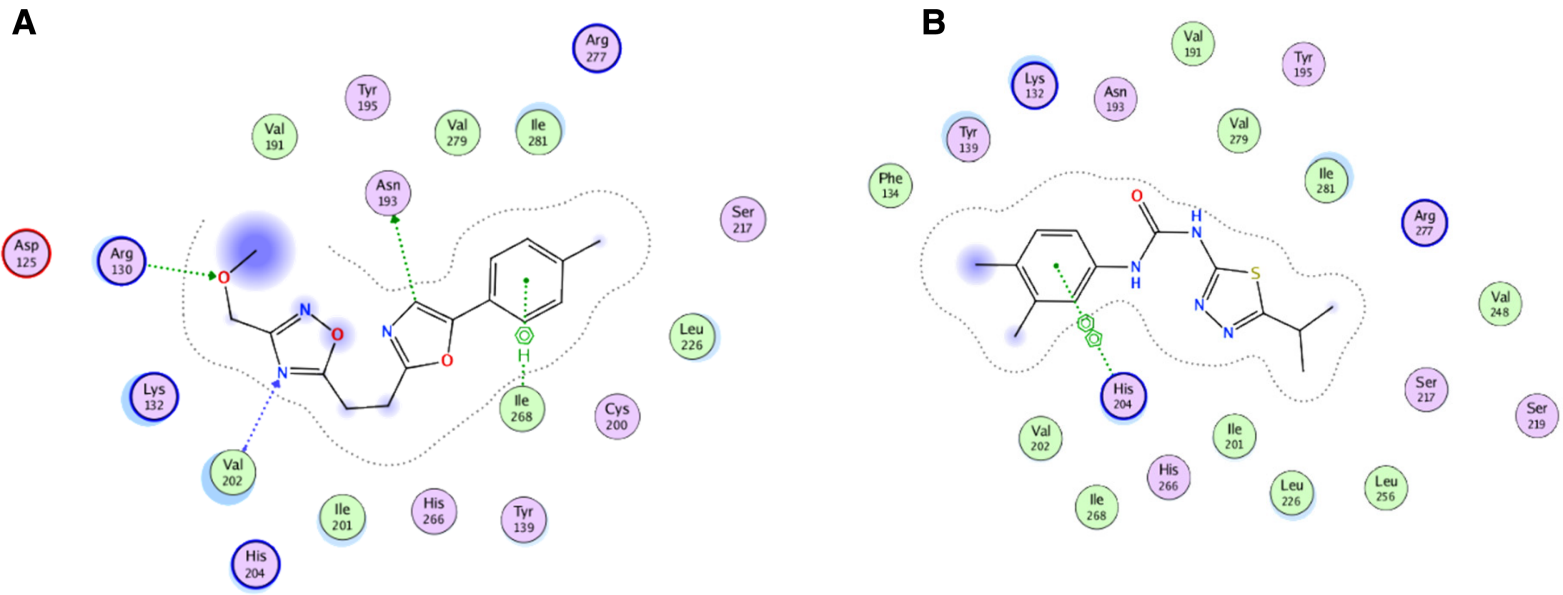
Two-dimensional (2D) representation of ligand-protein target interactions through the MOE. The top two best ranked docked compounds ZINC78774792 (**A**) and ZINC00546946 (**B**) in the most druggable cavity of *ALKBH5*.

**Figure 6:**
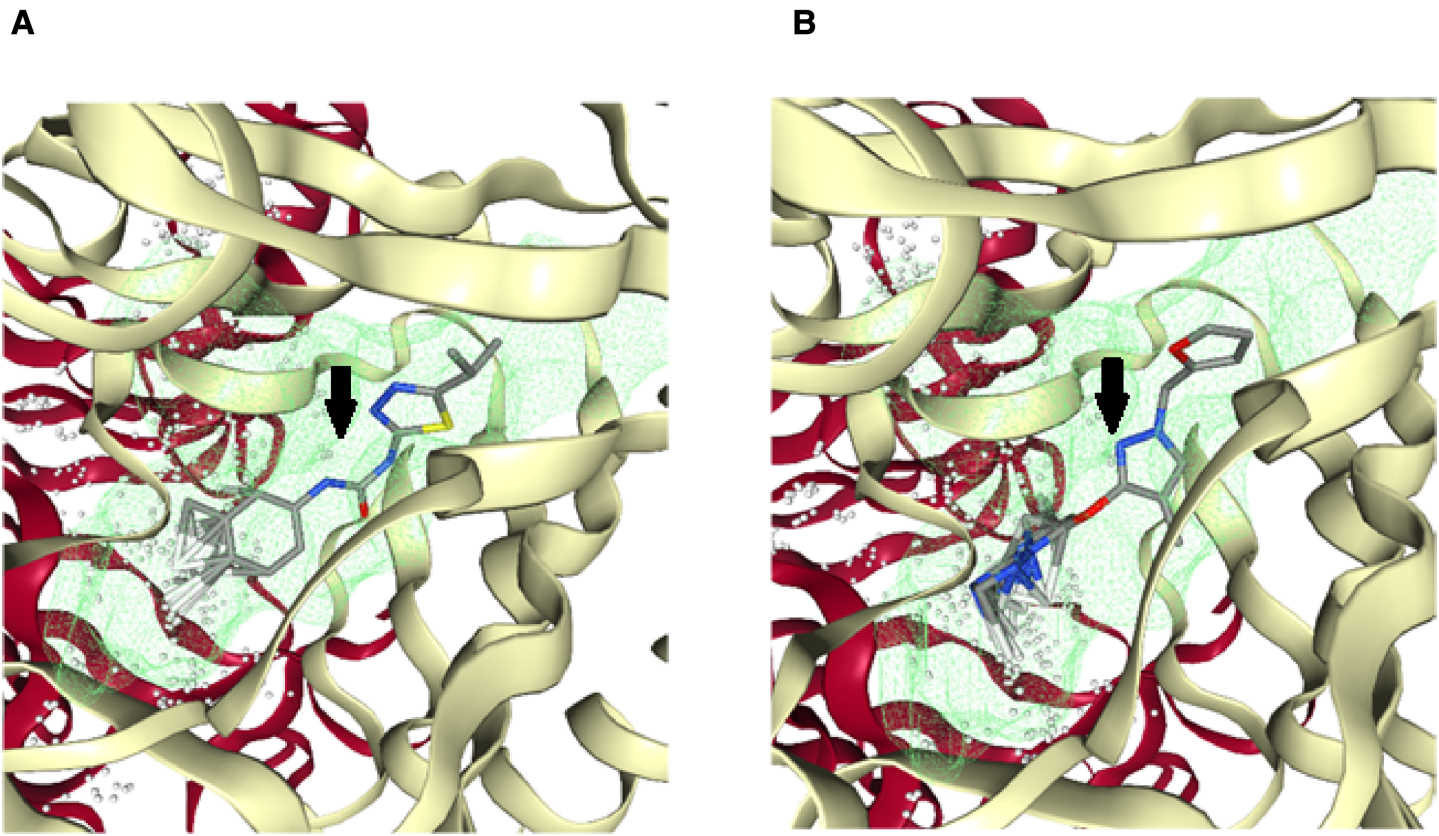
Three-dimensional (3D) cartoonic representation of the docked ligand-protein complexes through the DoGSiteScorer. The top 2 best ranked compounds ZINC78774792 (**A**) and ZINC00546946 (**B**) docked in the most druggable cavity of *ALKBH5*. The docked ligands have been pointed out through black arrows.

For the virtual screening using the 11,272 compounds (ZINC15 database) by em-ploying the MOE software, 5 best poses for each of the ligand molecules were recorded to find the best hit. The resulting list contained the best hits for the target protein *ALKBH5*. The interactions between the ligand and the active site of the protein (target-ligand complex structure) were evaluated to highlight the specific residues involved in the putative target activity. The lower energy scores of the MOE program indicates a better ligand–protein binding complex formation compared to high energy values. From the virtual screening, 10 compounds having the least binding energies (dG) with the *ALKBH5* were highlighted (Table 2). The lowest binding energy was found for ZINC78774792 compound (dG = −7.8428) which interacted with the Arg130, Asn193, Val202, and Ile268 residues of the active site of *ALKBH5* (Figure 5A). There was an arene-H interaction between the ligand and Ile268 residue, 1 proton-acceptor interac-tion with Asn293, and 2 proton-donor interactions between the ligand and Arg130 and Val202 residues. The second lowest binding energy was found for ZINC00546946 (Dg = −7.6338) which interacted with His204 residue (Figure 5B). There was an arene-arene interaction between the ligand and the His204 residue. Three dimensional (3D) repre-sentation of both the docked compounds in the most druggable cavity of *ALKBH5* through the DoGSiteScorer indicated the binding of both the compounds in the same orientation (Figure 6).

**Table 2:** Top 10 lead compounds shortlisted after the virtual screening of 11,272 compounds from the ZINC15 database, through the molecular docking to inhibit the *ALKBH5* protein. The structures of these compounds have been shown in Supplementary Table S8.

S. No	Compound	Score	Interacting residues
1	ZINC78774792	−7.8428	Arg130, Asn193, Val202, Ile268
2	ZINC00546946	−7.6338	His204
3	ZINC65397982	−7.6244	His 204
4	ZINC05114817	−7.5056	His204
5	ZINC40556313	−7.6061	Ile201
6	ZINC79110372	−7.4534	Ile201, Tyr139
7	ZINC40556313	−7.3501	Ile201
8	ZINC72270389	−7.3259	Lys132, Ile201
9	ZINC05602704	−7.2914	Tyr139
10	ZINC65507208	−7.2896	Ile201, His266

For the 10 selected compounds having the lowest binding energies, the ADMET properties (absorption, distribution, metabolism, excretion and toxicity) were studied to check their penetration and least side effects to human and other hosts, if any (Table 3). Out of the 10 short listed ligands, 5 molecules could cross the blood-brain-barrier, and 5 were found to be the substrates for P-glycoprotein (PGP). These compounds were found negative for causing mutagenicity in an Ames test, presumably indicating that these were not or least toxic to the host DNA replication or translation processes.

**Table 3:** Pharmacokinetic parameters of the top-scoring 10 ZINC compounds for the target *ALKBH5*. Pharmacokinetic parameters of 2 compounds (Ena15, and Ena21) from Takahashi et al. (35) also presented for comparisons.

S. No	Compound ID	Molar refractivity	Polar surface area topology (Å^2^)	Bioavailability	Blood brain barrier	PGP	Drug likeness Lipinski/violations	Lead likeliness/ violations	Consensus Log P o/w	Skin permeation Log Kp (cm/s)
1	ZINC78774792	79.88	71.48	0.55	Y	N	Y/0	Y/0	2.66	−6.31
2	ZINC00546946	83.14	95.15	0.55	N	N	Y/0	Y/0	3.01	−5.83
3	ZINC65397982	95.26	62.82	0.55	Y	Y	Y/0	Y/0	0.84	−7.52
4	ZINC05114817	90.64	98.83	0.55	N	Y	Y/0	Y/0	2.00	−6.81
5	ZINC40556313	78.32	69.79	0.55	Y	Y	Y/0	Y/0	3.21	−5.77
6	ZINC79110372	69.62	118.38	0.55	N	N	Y/0	Y/0	1.93	−6.33
7	ZINC40556313	78.32	69.79	0.55	Y	Y	Y/0	Y/0	3.21	−5.77
8	ZINC72270389	87.91	89.26	0.55	N	Y	Y/0	Y/0	2.11	−6.88
9	ZINC05602704	87.40	88.34	0.55	N	N	Y/0	Y/0	2.16	−6.02
10	ZINC65507208	76.07	67.60	0.55	Y	N	Y/0	Y/0	2.94	−5.60
11	Ena15	132.35	51.02	0.55	Y	Y	Y/0	N/3 violations: MW > 350, Rotors > 7, XLOGP3 > 3.5	4.59	−5.57
12	Ena21	87.36	48.95	0.55	Y	Y	Y/0	Y/0	3.00	−6.09

PGP, P-glycoprotein.

## 4. Discussion

Despite several investigations on cardiac dysfunction, the early diagnosis, treatment accuracy, and prognosis for cardiac failure remains unclear. Therefore, it is essential to explore the molecular mechanisms underlying the occurrence and progression of HF. In this study, we performed a series of bioinformatics analyses to explore the key genes and distinct expression profiles involved in the pathophysiology of HF in different age groups. The analysis was carried out on predominantly Caucasian individuals to minimize ethnicity-related bias. Despite several already identified genes in the HF were replicated in this analysis, a few novel genes, previously not or rarely reported in HF, were prioritized with considerable statistical significance (FDR < 0.05). These included the genes involved in platelet activation pathways, such as *CAP1, DGKD, F2R, FAM3C,* and *GNG12*, and genes of epitranscriptomics/epigenetics (Supplementary Table S7). Moreover, by applying a machine learning approach, we were able to find expression profiles related to various pathways in different ages of HF patients.

Among the genes of epitranscriptomics regulation, *ALKBH5* was found overexpressed in the HF. In a previous study, expression of *FTO* was found down-regulated causing an increase in global m^6^A in the HF due to ischemia compared with the normal heart (32). FTO-dependent N6-Methyladenosine regulates cardiac function during remodeling and repair (33). In the present study, which involves HF due to cardiomyopathy, no significant difference in the expression of *FTO* was observed between the HF and the normal (FDR > 0.05). Instead, the expression of *ALKBH5* was found to be increased in HF (FDR < 0.05). In this context, we may hypothesize that the underlying etiology of HF i.e., cardiomyopathy might have resulted in the difference in the regulation of epitranscriptomics. The over-expression of *ALKBH5* in the HF in the current analysis was found correlated with the significantly decreased global m^6^A methylation in the H9c2 cells (Figure 2A). The *ALKBH5-*induced global demethylation of RNAs may have caused altered splicing, degradation, and translation of the RNAs, as indicated previously (34), leading to the dysregulation of several cellular processes and resulting in HF. Collectively, these observations indicate a more likely role of *ALKBH5* in the pathophysiology of HF.

Having prioritized the *ALKBH5* through the *in silico* analysis and validated it through the estimation of global m^6^A level, ALKBH5 protein expression in H9c2 cells, we considered it a potential therapeutic agent for the treatment of HF. For this purpose, virtual screening (VS) of 11,272 drug-like compounds from the ZINC15 database was carried out in a molecular docking process using the MOE software, and the binding energies of the compounds with the *ALKBH5* protein (PDB ID 4NJ4) were determined. The VS of a large number of compounds (drug libraries) is an effective approach to reduce the cost and development time of new drugs. The resulting list of compounds contained the best-hits for the putative *ALKBH5* target. The 10 best-hit compounds short-listed in this study also passed the Lipinski's rule of five for the development of new drugs (31). The comparison of binding energies and ADMET parameters of the 10 best-hit compounds of this study with the Ena15 and Ena21 compounds, previously reported as potential inhibitors of *ALKBH5* (35), indicated that the binding energy of Ena15 and Ena21 in the *ALKBH5* active site was −7.3 kcal/mol, which was slightly higher than the binding energies of 6 out of 10 best-hit compounds found in this study (Table 2). In the ADMET properties, there were three violations from the Lead-likeliness rules for the Ena15 compound, whereas, the Ena21 and all the 10 best-hits of this study passed the Lead-likeliness rules. In this context, we can postulate that Ena21 and our 10 best-hits may be potential inhibitors of *ALKBH5*, and may be further implicated in drug development.

The epigenetic marker prioritized in this study *KMT2E* encodes a lysine methyltransferase 2E protein which is involved in chromatin remodeling. Previously, the role of *KMT2E* expression has been implicated in the prognosis of leukemias (36). However, the role of *KMT2E* in HF has not been reported yet. *KMT2E* can act as a potential novel drug target for HF after validating its cellular or organism models. The other genes found over-expressed in HF for the first time in this study included: *CAP1* which encodes the cyclase-associated actin cytoskeleton regulatory protein 1, which is an important regulator of actin cytoskeleton remodeling and signal transduction pathways (37). and *DGKD* encodes diacylglycerol kinase delta, a cytosolic enzyme that phosphorylates diacylglycerol to produce phosphatidic acid, and is associated with the response to elevated platelet cytosolic Ca^2+^ (38). Here, the over-expression of *DGKD* might be the result of calcium overload due to the Ca^2+^ release by the dense tubular system (DTS) and Ca^2+^ entry into the platelets from the extracellular space, resulting in disruption of haemostasis and vessel integrity in cardiac muscles (39). However, these hypotheses should be validated in animal models in subsequent studies.

Cardiac physiology alters with the passage of age, and studies have suggested that age is an intrinsically a major independent risk factor for CVDs. For example, in various genetically modified aged mice models, the role of adrenergic and renin-angiotensin II signaling, insulin/insulin-like growth factor/PI3K pathway, mitochondrial oxidative stress, and nutrient signaling pathways were observed (40). However, incidences of HF are observed in the elderly as well as in the early ages. Here, we stratified the genes expression profiles in the HF of young-aged adults (average 23.33 years), late-aged adults (∼43 years), and early-aged elders (∼57.66 years) patients. These profiles indicated the likely distinct mechanisms underlying in the HF of different age groups. In young-aged adult patients, the pathways of energy homeostasis [Valine, leucine and isoleucine degradation (hsa00280), and 2-Oxocarboxylic acid metabolism (hsa01210)] were found activated indicating metabolic remodeling in the cardiac tissue and highly likely imparting a part to HF, which correlates with previous reports (41). In late-aged adult HF patients, the genes which are involved in thyroid hormone signaling pathway (hsa04919) and aldosterone-regulated sodium reabsorption pathway (hsa04960) were found predominantly over-expressed in the heart tissue. These pathways have been linked with the higher blood pressure (42). In the early-aged elders of HF, the genes involved in the activation of inflammatory processes, neutrophil chemotaxis, and actin filament organization were found over-expressed. While immune cell infiltrations have been well described in HF (43), recently, the role of actin filament organization has also been described in HF (44). Collectively, these findings suggest that distinct molecular mechanisms operate in the cardiac tissue and disrupt vital cellular processes leading to HF.

## 5. Conclusion

Given the complex pathophysiology of HF, multiple molecular etiologies impart their roles in the onset of this disease. This study identified novel markers including an epitranscriptomics gene *ALKBH5* being over-expressed in the HF. By using the molecular docking, two ZINC15 compounds (ZINC78774792, and ZINC00546946) were prioritized as potential inhibitors which bound in the *ALKBH5* active site with the lowest binding energies. Additionally, distinct gene expression profiles in the HF of young-aged adults (∼23 years), late-aged adults (∼43 years), and early-aged elders (∼57.6 years) were identified by employing the unsupervised hierarchal clustering. Additionally, the genes of actin cytoskeleton regulation (hsa04810), focal adhesion (hsa04510), and PI3K-Akt signaling pathway (hsa04151) were having lower expression in male heart failures compared with females. These preliminary findings of the study identified some of molecular mechanisms involved in heart failure. We suggest to validate these results in animal models to obtain definitive conclusions. Furthermore, the identified compounds may have potential for treating HF, and further investigations using animal models could evaluate their efficacy.

## Data Availability

The datasets presented in this study can be found in online repositories. The names of the repository/repositories and accession number(s) can be found in the article/**Supplementary Material**.
