# Supplementary material for: ALKBH5 inhibitors as a potential treatment strategy in heart failure—inferences from gene expression profiling
Source: Front Cardiovasc Med. 2023 Jul 31;10:1194311. doi: 10.3389/fcvm.2023.1194311 (PMC10425272; doi:10.3389/fcvm.2023.1194311)
Supplement: Supplementary file 1 [file Datasheet1.docx]

**Supplementary Figures**


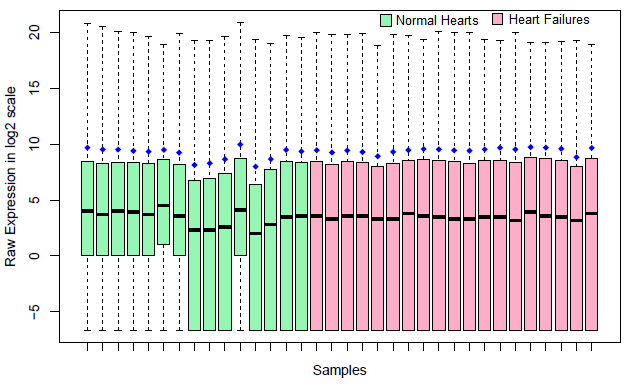


**Supplementary Figure S1:** Box plots indicating the ranges of FPKM values in the Normal and Heart Failure samples. The blue dots represent average FPKM, and the middle lines in the boxes represent median FPKM.


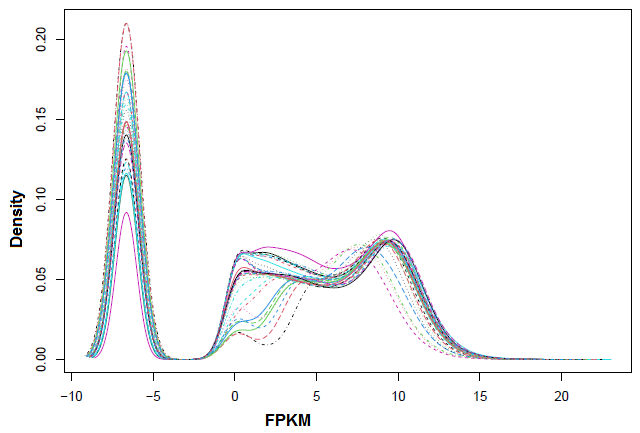


**Supplementary Figure S2:** Distribution of FPKM values in the study samples. The differences in the FPKM distribution among the samples are observable at FPKM values 0-5, and 8-14.


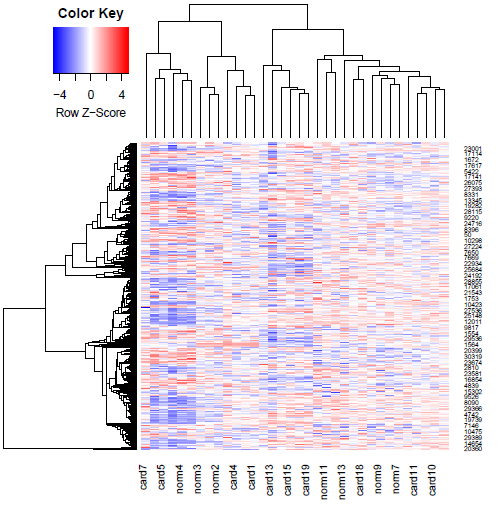


**Supplementary Figure S3:** A heatmap showing the genes expression pattern based on FPKM values, prior to the differential expression of genes analysis. The control, and cardiac samples are found mixed together.


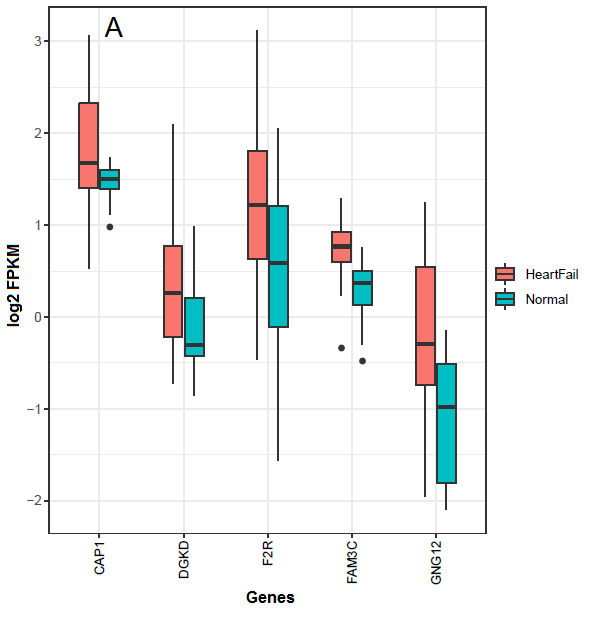


**Supplementary Figure S4:** Significantly differentially expressed novel genes related with platelets activation and aggregation pathway in the heart failure compared with the normal hearts.


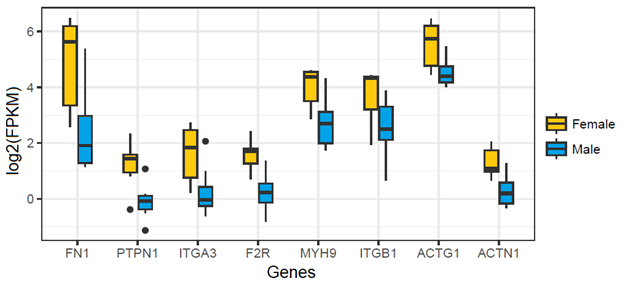


**Supplementary Figure S5:** Sex-wise differential expression in the heart failures. The FPKM values of 8 genes significantly over-expressed in the female heart failures compared with those in males.
