# Supplementary material for: ALKBH5 inhibitors as a potential treatment strategy in heart failure—inferences from gene expression profiling
Source: Front Cardiovasc Med. 2023 Jul 31;10:1194311. doi: 10.3389/fcvm.2023.1194311 (PMC10425272; doi:10.3389/fcvm.2023.1194311)
Supplement: Supplementary file 9 [file Table8.docx]

**Supplementary Table S9:** Zinc database IDs and structures of the Alkbh5 inhibitors presented in this study.

| **Compound ID** | **SMILES** | **Structure** |
| --- | --- | --- |
| ZINC78774792 | COCc3noc(CCc2ncc(c1ccc(C)cc1)o2)n3 | 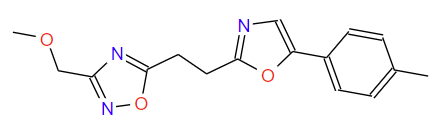 |
| ZINC00546946 | Cc2ccc(NC(=O)Nc1nnc(C(C)C)s1)cc2C | 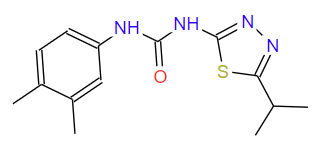 |
| ZINC65397982 | CCCn3cc(NC(=O)c2cccc(C[N+]1=CCCC1)c2)nn3 | 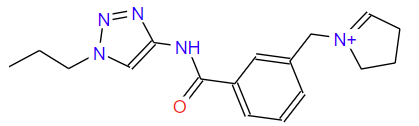 |
| ZINC05114817 | CCOC(=O)Nc3cc2OCC(c1ccccc1)=Nc2c(N)n3 | 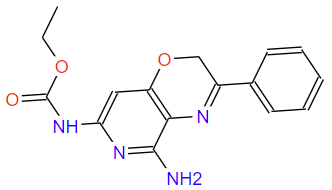 |
| ZINC40556313 | Cc3nc(CSc2ccc1OCCCOc1c2)oc3C | 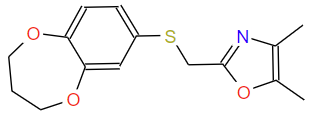 |
| ZINC79110372 | CCCc2noc(COC(=O)c1cnc(NC)s1)n2 | 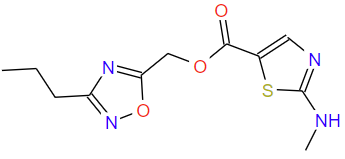 |
| ZINC72270389 | C#CCN1CCC[C@H]1C(=O)Nc3cc(c2ccsc2)n[nH]3 | 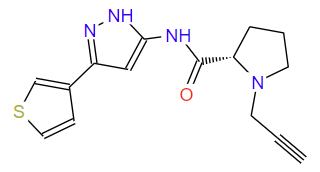 |
| ZINC05602704 | O=C(NNp2[nH]nc(c1ccccc1)o2)c3ccccc3 | 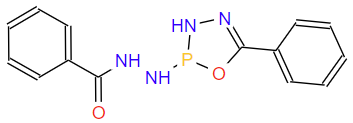 |
| ZINC65507208 | CCc3nc(C)c(c2nc(Cc1ccccc1)n[nH]2)o3 | 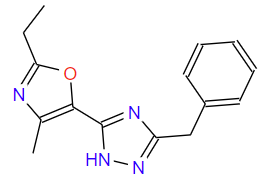 |
